# An Unusual Presentation of Cobblestone Esophagus From Bisphosphonate Use

**DOI:** 10.7759/cureus.52602

**Published:** 2024-01-19

**Authors:** Tian Yu Qiu, Yi Yuan Tan, Nicholas Tee Chin Hock, Rajesh R

**Affiliations:** 1 Gastroenterology and Hepatology, Singapore General Hospital, Singapore, SGP; 2 Gastroenterology and Hepatology, Changi General Hospital, Singapore, SGP

**Keywords:** alendronate, asymptomatic esophagitis, bisphosphonate, drug-induced esophagitis, cobblestone esophagus

## Abstract

Cobblestone esophagus is a rare finding that has been previously described in cases of eosinophilic esophagitis (EoE), *candidiasis*, Barrett’s esophagus, or severe reflux esophagitis from distal gastrointestinal obstruction. We describe a case of asymptomatic cobblestone esophagus secondary to bisphosphonate use.

A 67-year-old female was seen in the clinic for evaluation of microcytic anemia that was incidentally picked up on routine chronic disease follow-up. She had no gastrointestinal symptoms. She has been taking oral alendronate 70mg once a week for osteoporosis since a year ago. Barium meal was performed as the patient initially opted for non-invasive testing, which incidentally showed a diffuse “cobblestone” appearance. Subsequent oesophago-gastro-duodenoscopy (OGD) showed diffuse white nodular lesions along the esophagus with a cobblestone appearance but no ulcer or mass. Segmental esophageal biopsies were negative for fungal stain and did not show any pathology. In the absence of infection, eosinophilic esophagitis, and dysplasia, her “cobblestone” esophagus was attributed to bisphosphonate use by diagnosis of exclusion. Alendronate acid was held off, and serial barium meals over the next year showed significant interval improvement.

Bisphosphonates, such as alendronate acid, are commonly associated with drug-induced esophagitis. With the cessation of the offending medication, there was indeed a significant improvement in our patient’s serial barium meal. It is important to review the medication list when encountering patients who present with cobblestone esophagus, as some of these patients with drug-induced esophagitis may be asymptomatic clinically.

## Introduction

Cobblestone esophagus is a rare finding that has been previously described in cases of eosinophilic esophagitis (EoE), *candidiasis*, Barrett’s esophagus, or severe reflux esophagitis from distal gastrointestinal obstruction [1-4]. We describe an unusual case of asymptomatic cobblestone esophagus secondary to bisphosphonate use.

## Case presentation

Clinical situation

A 67-year-old female presented to our outpatient clinic for evaluation of iron deficiency anemia that was incidentally picked up on routine chronic disease follow-up. Her past medical history includes hypertension, type 2 diabetes mellitus, hypothyroidism, and osteoporosis. She had dyspepsia but had no other symptoms; in particular, she did not have any dysphagia, odynophagia, or reflux symptoms. She was started on oral alendronate once a week a year ago when she was diagnosed with osteoporosis after a compression fracture. Her other medications included telmisartan, iron polymaltose, cholecalciferol capsules, and amlodipine. 

Course of events

**Figure 1:**
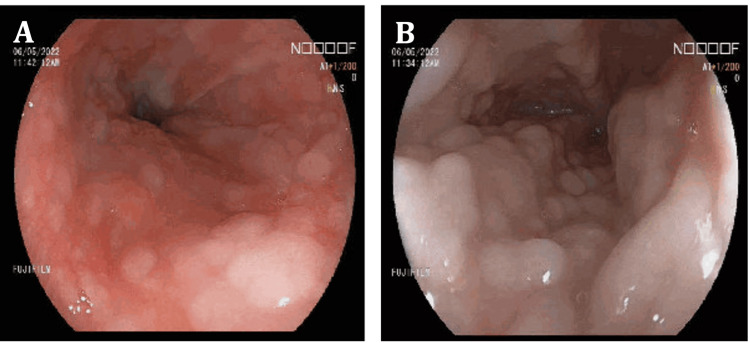
Endoscopic view of the esophagus (A and B), which showed diffused white nodular lesions along the esophagus with cobblestone appearance; no ulcer or mass was noted.

She was initially only keen for non-invasive tests; hence, a barium meal was performed, which showed a diffuse “cobblestone” appearance but no stricture or significant gastro-esophageal reflux. She subsequently agreed for oesophago-gastro-duodenoscopy (OGD), which showed diffused white nodular lesions along the esophagus starting from the oropharynx with a cobblestone appearance, but no ulcer or mass noted (Figure 1). Segmental esophageal biopsies were negative for fungal stain and did not show any pathology. In the absence of infection, eosinophilic esophagitis, and dysplasia, her “Cobblestone” esophagus was attributed to bisphosphate use; hence, alendronate acid was held off.

Clinical resolution

**Figure 2:**
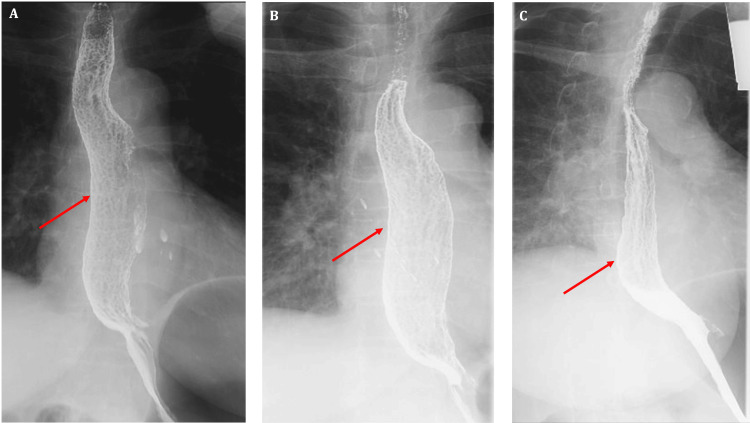
Serial barium meals performed (A) at diagnosis, (B) after one year of bisphosphonate cessation, and (C) after two years of bisphosphonate cessation

She underwent serial barium meals over the next year with significant interval improvement (Figure 2).

## Discussion

Cobblestone esophagus is an uncommon finding on endoscopy that has been associated with conditions like EoE and candidiasis, but these causes were excluded in our patient during the endoscopy and biopsy. By diagnosis of exclusion, alendronate acid use was deemed the most likely cause of this patient’s cobblestone esophagus, although no similar case has been reported before in the literature.

Bisphosphonates, such as alendronate acid, are commonly associated with drug-induced esophagitis [5] and can cause esophageal irritation by breaching the protective hydrophobic mucosal lining [6-7]. The cobblestone pattern observed during barium meal and endoscopy is a result of inflammation and edema of the lamina propria and submucosal layers over time [8]. In our patient, the biopsy did not show this, but this could be due to superficial sampling of the mucosa alone. The mainstay of treatment for drug-induced esophagitis is the cessation of the offending medication [9]. There was indeed a significant improvement in serial barium meal in our patient upon cessation of alendronate acid. During this period, she was not given any proton pump inhibitors either. As for her osteoporosis, she was started on subcutaneous denosumab and continued on this without any issues. This underscores the importance of reviewing the medication list when encountering patients exhibiting cobblestone esophagus, in addition to ruling out the known underlying causes mentioned earlier.

The clinical challenge of this case lies in its atypical, asymptomatic presentation of drug-induced esophagitis. Patients with drug-induced esophagitis frequently complain of symptoms such as retrosternal chest pain, which happens in about 70% of the patients, as well as odynophagia and dysphagia [9]. In our patient, it was first picked up incidentally on barium meal as part of anemia workup before confirming it with endoscopy. With this in mind, it is important to counsel our patients on the correct administration of bisphosphonate [10] so as to minimize upper gastrointestinal adverse effects that may initially be asymptomatic and delay timely action before long-term side effects are observed.

## Conclusions

The finding of a cobblestone esophagus should prompt physicians to investigate for an underlying etiology. In addition to performing endoscopies and biopsies to exclude common etiologies, this case shows us the importance of taking a comprehensive drug history. Patients with drug-induced esophagitis may be asymptomatic initially. It is an easily reversible etiology that should be routinely considered for prompt intervention against irreversible damage to the upper gastrointestinal tract.
